# Intraspinal hematoma caused by anticoagulant rodenticide poisoning: a rare case report

**DOI:** 10.3389/fmed.2026.1795706

**Published:** 2026-04-13

**Authors:** Wei Jie, Ting Liu, Miao Dai

**Affiliations:** 1 Department of Geriatrics, Jiujiang NO.1 People’s Hospital, Jiujiang, Jiangxi, China; 2 Department of Cardiovascular and Neurology, Jiujiang Sixth People's Hospital, Jiujiang, Jiangxi, China; 3 Chronic Disease Management Center, Jiujiang NO.1 People’s Hospital, Jiujiang, Jiangxi, China

**Keywords:** anticoagulant rodenticide, coagulopathy, intraspinal hematoma, poisoning, superwarfarin

## Abstract

**Introduction:**

Anticoagulant rodenticide poisoning is a significant public health issue, particularly in rural areas. Common clinical manifestations include mucocutaneous bleeding, hematuria, gastrointestinal bleeding, and ecchymosis. While intracranial hemorrhage is a recognized and serious complication, the occurrence of an intraspinal hematoma (ISH) secondary to such poisoning is exceptionally rare, with no well-documented cases in the existing literature.

**Case presentation:**

We report the case of a 78-year-old male farmer who presented with severe right lower limb pain of 5 days’ duration. His history revealed ingestion of an unknown rodenticide approximately 1 month prior, followed by episodes of hematuria and hematemesis that had been partially treated with vitamin K1. Upon admission, he exhibited a severe coagulopathy with markedly elevated prothrombin time and International Normalized Ratio. Magnetic resonance imaging of the lumbar spine revealed an intraspinal mass at the L1 level, which was subsequently confirmed to be a hematoma on a contrast-enhanced study. The patient was diagnosed with an intraspinal hematoma complicating SUPERWARFARIN poisoning. Management included the administration of vitamin K1 and fresh frozen plasma, leading to a gradual normalization of coagulation parameters and symptomatic improvement.

**Conclusion:**

This case highlights a highly unusual and severe neurological complication of presumed anticoagulant rodenticide poisoning. It underscores the critical importance of maintaining a high index of suspicion for hemorrhagic events in unusual anatomical locations in patients with a history of rodenticide exposure and unexplained coagulopathy.

## Introduction

Anticoagulant rodenticides-often referred to as “superwarfarins” because of their greater potency and longer duration of action relative to the therapeutic agent warfarin—are a class of compounds widely used for pest control. Despite existing regulations, accidental or intentional ingestion continues to pose a persistent clinical challenge, especially in agricultural and rural communities where access to these substances may be less restricted (1). The toxicological mechanism of these compounds is well established: they act as potent antagonists of vitamin K epoxide reductase, a key enzyme in the hepatic synthesis of vitamin K-dependent clotting factors (II, VII, IX, and X), as well as the natural anticoagulants protein C and protein S (2). This inhibition leads to the production of functionally inactive, partially carboxylated clotting factors, resulting in a profound state of coagulopathy.

The clinical presentation of anticoagulant rodenticide poisoning typically includes a latent period, ranging from 24 h to several days or even weeks following ingestion, as the body depletes its existing stores of functional clotting factors (3). Bleeding manifestations are diverse and may involve virtually any organ system. Common clinical findings include easy bruising, gingival bleeding, epistaxis, hematuria, gastrointestinal bleeding (presenting as hematemesis or melena), cerebral hemorrhage, and, in women, menorrhagia (4–6). More severe, life-threatening complications involve internal bleeding-such as retroperitoneal hematomas and, most notably, intracranial hemorrhage (ICH) (6–8) which is a leading cause of mortality and morbidity in these patients.

Although coagulopathy is a well-recognized risk factor for spinal hematomas-especially in the setting of trauma, lumbar puncture, or therapeutic anticoagulation-its occurrence as a direct and primary consequence of rodenticide poisoning appears to be unprecedented. This case report, therefore, documents a unique and severe neurological complication in a patient with highly suspected anticoagulant rodenticide toxicity, emphasizing the need for expanded clinical vigilance.

## Case presentation

A 78-year-old male farmer presented to the hospital with a five-day history of severe, persistent pain in the right lower limb. The pain began on August 17, 2024, in the absence of any identifiable trauma or precipitating event. He described the sensation as a “pulling” or “tugging” originating from the lateral aspect of the right thigh, radiating along the distribution of the sciatic nerve to the calf and specifically to the second toe. The pain was constant and severe, markedly limiting his ability to ambulate. Moreover, it was debilitating enough to impair his appetite and disrupt nocturnal sleep. At the time of presentation, he reported no associated numbness, tingling, or weakness, and denied any back pain or bowel or bladder incontinence.

The patient’s history was significant for a recent episode of coagulopathy. Approximately 1 month prior to the current admission (early July 2024), he had accidentally ingested a rodenticide used for pest control on his farm. He was unable to specify the brand name or the exact active ingredient of the ingested substance, as he did not retain the packaging. About 2 weeks after ingestion, he developed painless gross hematuria and episodes of hematemesis. He was treated with intravenous vitamin K1 (specific dose not documented in referral records) for 11 days, after which the hematuria and hematemesis resolved. Following discharge, he received a daily 10 mg intramuscular dose of vitamin K1 at a local clinic for approximately 2 weeks. A few days prior to the current presentation, he noted the appearance of mild subcutaneous bruising over his left ankle, signaling a recurrence of coagulopathy.

**Figure 1:**
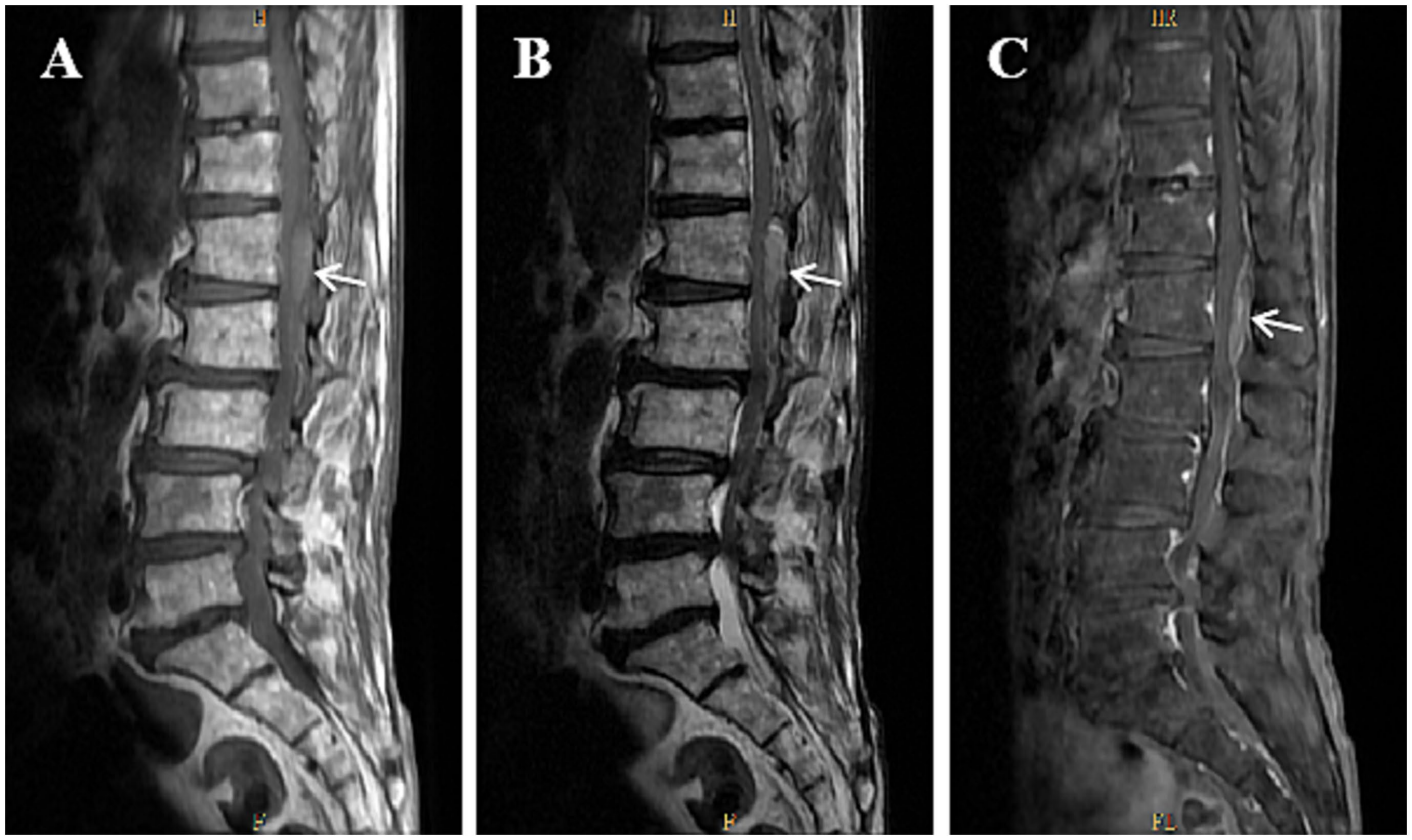
Magnetic resonance imaging (MRI) of the lumbar spine at the L1 level. **(A)** Sagittal T1-weighted image shows a 29 × 9 mm spindle-shaped, slightly hyperintense lesion within the spinal canal (white arrow). No abnormal paraspinal soft tissue signal is noted. **(B)** Sagittal T2-weighted image demonstrates the lesion with similar morphology and slightly hyperintense signal (white arrow). **(C)** Contrast-enhanced T1-weighted image reveals no significant enhancement of the lesion, suggestive of a hematoma (white arrow).

On admission, spinal examination revealed kyphosis but no localized tenderness or percussion pain. Neurological evaluation was notable for a positive straight leg raise test on the right side, suggestive of nerve root irritation or compression. Muscle strength and tone were normal in all four limbs. Pathological reflexes and meningeal signs were absent. No peripheral edema or joint abnormalities were observed. Initial blood tests showed normocytic, normochromic anemia (hemoglobin: 103 g/L, red blood cell count: 3.48 × 10^12^/L). White blood cell and platelet counts were within normal limits. The most striking abnormalities were noted in the coagulation profile. Renal function, electrolytes, and cardiac enzymes were within normal ranges. C-reactive protein was not significantly elevated (0.90 mg/dL) (Table 1). Lumbar MRI (plain scan, August 18, 2024) revealed degenerative changes in the lumbar spine, including L3 spondylolisthesis and disc bulging from L2 to S1. Importantly, it identified a space-occupying lesion within the spinal canal at the L1 level (Figures 1A,B). Lumbar MRI with contrast (August 20, 2024) confirmed the presence of the L1 intraspinal mass. The lesion did not show significant contrast enhancement, leading to a radiological impression most consistent with a hematoma (Figure 1C).

**Table 1:** Clinical and laboratory variables.

Blood analytes	August 17th, 2024	August 18th, 2024	September 2th, 2024	Normal value
White blood cell	5.5	–		3.5–9.5 × 10^9^/L
Neutrophil	3.5	–		1.8–6.3 × 10^9^/L
Haemoglobin	103.0	–		130–175 g/L
Platelets	155.0	–		125–350 × 10^9^/L
C-reactive protein	0.9	–		0–0.6 mg/dL
D-dimer	0.3	0.3	0.2	0–1.0 mg/L(FEU)
INR	10.05	1.52	1.50	0.86–1.20
APTT	106.0	38.7	30.4	24–34 s
Fibrinogen	3.8	3.4	5.96	2-4 g/L
Thrombin time	18.5	19.8	19	14–24 s

INR, International standardization ratio; APTT, activated partial thromboplastin time.

The patient’s management focused on two primary objectives: reversing the life-threatening coagulopathy and addressing the symptomatic spinal lesion. He received a single unit (200 mL) of virus-inactivated fresh frozen plasma (blood group A+) for immediate replacement of clotting factors. Concurrently, high-dose intravenous vitamin K1 (40 mg administered as a slow infusion daily for 5 days) was initiated to restore endogenous synthesis of functional clotting factors. Given the location and nature of the intraspinal hematoma, along with the patient’s stable neurological status (absence of motor deficits), a conservative, non-surgical approach was adopted, and close neurological monitoring was implemented.

The patient showed a favorable response to the coagulation reversal therapy. Follow-up coagulation studies demonstrated a steady normalization of PT and INR. At the time of discharge, the patient’s radicular pain had markedly improved (visual analog scale score reduced from 8/10 to 2/10), and motor strength remained intact. He was discharged with a plan for prolonged oral vitamin K1 therapy at a dose of 20 mg three times daily. Given the long half-life of superwarfarins, the patient and his family were counseled that this regimen was expected to continue for several months, with strict instructions for regular monitoring of his coagulation parameters (prothrombin time/INR) on a weekly to bi-weekly basis to guide eventual dose tapering and prevent recurrence. Two month after discharge, telephone follow-up revealed complete resolution of the right lower limb pain. The patient reported that a follow-up lumbar MRI performed locally showed complete resolution of the intraspinal hematoma; however, the imaging files and formal radiology report were not available for review. No recurrent bleeding events were reported.

## Discussion

We present a rare and instructive case of a 78-year-old male who developed an intraspinal hematoma, a highly unusual complication secondary to likely poisoning from a long-acting anticoagulant rodenticide (LAAR). This case underscores the profound and systemic impact of LAARs, highlights the challenges in their long-term management, and serves as a critical reminder to clinicians to consider hemorrhagic complications in virtually any anatomical location when presented with a history of such poisoning.

The patient’s clinical course is a classic, albeit severe, presentation of LAAR toxicity. The timeline is highly suggestive: an initial ingestion around early July 2024, followed by a latent period of approximately 2 weeks before the manifestation of gross hematuria and hematemesis. This delayed onset is a well-documented characteristic of LAARs like brodifacoum, which act by inhibiting the vitamin K epoxide reductase complex, thereby preventing the recycling of vitamin K and the subsequent synthesis of active clotting factors II, VII, IX, and X. The depletion of these factors, particularly factor VII with its short half-life, leads to a progressive coagulopathy that becomes clinically apparent only after a significant delay (9). The initial response to parenteral vitamin K1 therapy, followed by a relapse upon its presumed discontinuation or inadequate dosing at a local clinic, further solidifies this diagnosis. The profound coagulopathy upon admission, evidenced by a prothrombin time (PT) of 106.7 s and an International Normalized Ratio (INR) of 10.05, confirms a severe state of anticoagulation.

The central and most remarkable feature of this case is the development of an intraspinal hematoma at the L1 level. While hemorrhagic diatheses from LAAR poisoning commonly manifest as mucocutaneous bleeding (e.g., ecchymosis, gingival bleeding), gastrointestinal hemorrhage, hematuria, or retroperitoneal hematomas (10), bleeding into the central nervous system (CNS) is a feared but less common event. The majority of reported CNS hemorrhages associated with LAARs involve intracranial bleeding (6, 11), which can be life-threatening. In contrast, an isolated intraspinal hematoma is an exceptionally rare complication. The precise localization of the intraspinal hematoma at the L1 level in this patient warrants consideration of potential local predisposing factors beyond the systemic coagulopathy. While the most pronounced degenerative changes-L3 spondylolisthesis and L2-S1 disc herniation-were not at the exact level of the bleed, the patient exhibited generalized lumbar spondylosis. Such degenerative changes can compromise the integrity of the internal vertebral venous plexus (Batson’s plexus), which is a low-pressure, valveless network that is vulnerable to rupture under conditions of altered hemostasis. Chronic mechanical stress from instability, osteophytes, or disc disease can lead to epidural venous engorgement or microvascular fragility. In the context of profound coagulopathy, as evidenced by an INR of 10.05, a spontaneous rupture of such a compromised venous structure may occur. Unlike iatrogenic spinal hematomas, which are often associated with lumbar puncture or epidural anesthesia, or those linked to vascular malformations, this case likely represents a multifactorial etiology. The severe coagulopathy served as the primary systemic trigger, while the patient’s underlying degenerative spinal disease likely created a site-specific anatomical vulnerability. This interaction between systemic and local factors is well-recognized in spontaneous spinal hematomas associated with therapeutic anticoagulation but has not been previously described in the context of rodenticide poisoning. This case suggests that in patients with superwarfarin-induced coagulopathy, the presence of even subclinical spinal degeneration may represent a risk factor for the development of a spontaneous intraspinal hematoma (ISH).

A review of the existing medical literature reveals a stark scarcity of reported cases linking anticoagulant rodenticides to intraspinal hematomas. Most case series and reviews on LAAR poisoning complications focus extensively on intracranial hemorrhage (ICH). For instance, a comprehensive review by King and Tran (12) analyzing 174 cases of superwarfarin poisoning identified intracranial hemorrhage in 5.7% of patients, but made no mention of spinal hematomas. The pathophysiological reason for this disparity is not entirely clear but may relate to anatomical and hemodynamic factors. The cerebral vasculature is extensive and, in older individuals, may be more susceptible to microtrauma or underlying vascular anomalies like cerebral amyloid angiopathy. In contrast, the spinal canal is a relatively protected space with a robust ligamentous support system and a different vascular architecture. Spontaneous spinal hematomas are most frequently associated with arteriovenous malformations, tumors, or iatrogenic causes (e.g., lumbar puncture, epidural anesthesia), with coagulopathies being a less common, but recognized, predisposing factor. When coagulopathy is the cause, it is typically due to therapeutic anticoagulation (e.g., warfarin, heparin) or hematological malignancies, not rodenticide poisoning.

The diagnostic journey in this case was pivotal. The initial presentation of lower limb pain could have been misattributed solely to the evident lumbar disc disease and degenerative changes seen on MRI. However, the patient’s history of rodenticide poisoning and the presence of new ecchymosis raised the suspicion of a coagulopathy-related complication. The initial non-contrast MRI identified the “space-occupying lesion,” and the subsequent contrast-enhanced scan appropriately suggested a hematoma, correctly guiding the clinical team away from a primary tumor or other pathology. This step-wise imaging approach, firmly contextualized by the clinical history, was crucial for accurate diagnosis and prevented unnecessary invasive procedures.

The management of this patient aligns with established principles for LAAR poisoning but is complicated by the critical location of the bleed. The acute reversal of coagulopathy was achieved with virus-inactivated fresh frozen plasma, providing immediate replacement of clotting factors. Simultaneously, high-dose intravenous vitamin K1 (40 mg daily) was initiated to restore the body’s intrinsic synthesis of these factors. This dual approach is standard for severe or life-threatening bleeding. The challenge with LAARs, however, lies in the extraordinarily long half-life of these compounds, which can persist in the body for weeks to months. This necessitates a prolonged course of high-dose vitamin K1 therapy, often for several months, to prevent rebound coagulopathy once the exogenous plasma factors are cleared. As highlighted by Yip et al., the median treatment duration for brodifacoum poisoning can exceed 100 days (9). The transient improvement in coagulation parameters following the 5-day course of intravenous therapy, and the need for a continued high-dose oral regimen, exemplifies this phenomenon. The patient’s relapse after the initial 11-day course of intravenous vitamin K1 and subsequent short course of intramuscular therapy serves as a critical teaching point, underscoring that inadequate dosing or premature discontinuation of therapy is a common pitfall in the management of LAAR poisoning. Our discharge plan, consisting of a prolonged high-dose oral vitamin K1 regimen with close outpatient monitoring, aligns with the recommended long-term management strategy to prevent recurrence (9).

A significant limitation of this report is the absence of confirmatory toxicological testing for specific superwarfarins (e.g., serum or urine concentrations of brodifacoum or bromadiolone). While such testing represents the gold standard for definitive diagnosis, it was not available in our clinical setting. The diagnosis of long-acting anticoagulant rodenticide (LAAR) poisoning in this case is therefore presumptive and based on a constellation of compelling clinical features: a clear history of accidental rodenticide ingestion, the classic latent period preceding hemorrhagic manifestations, a profound vitamin K-dependent coagulopathy (evidenced by a markedly elevated INR), a prompt and sustained response to high-dose vitamin K1 therapy, and the exclusion of other common etiologies such as liver disease (normal liver function tests) and therapeutic anticoagulation. We acknowledge this limitation and emphasize that while the diagnosis is highly probable, it is not definitively confirmed by toxicological analysis.

In conclusion, this case provides several critical lessons for clinical practice. First, it expands the spectrum of possible hemorrhagic complications of LAAR poisoning to include the rare but serious possibility of an intraspinal hematoma. Clinicians must maintain a high index of suspicion for bleeding in any organ system, including the spinal canal, in patients with a history of superwarfarin exposure and new neurological symptoms. Second, it reinforces the principle that management of LAAR toxicity is a marathon, not a sprint. Inadequate dosing or premature discontinuation of vitamin K1 can lead to relapse and severe complications, as seen here. Third, where available, toxicological investigation should be considered to confirm the specific rodenticide involved, as certain compounds may persist in the body for weeks to months and guide the duration of therapy. Finally, this case highlights the importance of a thorough social and medical history, as the key to the diagnosis was the patient’s reported ingestion of a banned substance weeks prior. As these rodenticides remain a public health concern, particularly in rural areas, physicians worldwide must be equipped to recognize and manage their diverse and potentially devastating clinical presentations.

## Data Availability

The raw data supporting the conclusions of this article will be made available by the authors, without undue reservation.
